# Endobronchial metastases secondary to prostate cancer: A case report and literature review

**DOI:** 10.1016/j.rmcr.2020.101326

**Published:** 2020-12-29

**Authors:** Mansoor Hameed, Irfan Ul Haq, Muhammad Yousaf, Mousa Hussein, Umar Rashid, Issam Al-Bozom

**Affiliations:** a Hamad General Hospital, Hamad Medical Corporation, Doha, Qatar; b Hazm Mebaireek Hospital, Hamad Medical Corporation, Doha, Qatar; c Weill Cornell Medicine-Qatar, Cornell University, NY, USA

**Keywords:** Endobronchial metastases, Prostate cancer, Extrapulmonary tumours

## Abstract

Metastatic disease from solid extrapulmonary tumours affects the lungs frequently. Endobronchial metastases (EM) however are very rare. Most commonly breast, colorectal and renal carcinomas can result in endobronchial metastases. EM secondary to a prostate primary are even more uncommon. We present an unusual case of a synchronous diagnosis of EM and primary prostate cancer. The diagnosis was confirmed on bronchoscopic endobronchial biopsies and immunohistochemical examination. Just 3 such cases have been reported to the best of our knowledge in the last 15 years. We discuss frequencies, similarities with previously reported cases, possible developmental modes and the diagnosis of EM. We conclude that patients with a current or previous diagnosis of an extrapulmonary malignancy with apparently trivial respiratory symptoms and/or unexplained weight loss should be considered for a bronchoscopy. Bronchoscopy and immunohistochemical profiling is the gold standard for diagnosing EM, as they may not be visible on cross sectional imaging.

## 1 Introduction

Lungs are one of the commonest sites of metastases from solid extrapulmonary malignancies, with an incidence of around 20%–54% [1]. Such metastases normally involve the lung parenchyma, pleura or adjacent structures. Endobronchial metastases (EM) however, are a rare occurrence. The most common tumours leading to endobronchial metastases are breast, colorectal and renal carcinomas [2,3].

Prostate cancer is a commonly seen malignancy in men, which can lead to skeletal, nodal or pulmonary metastases. Endobronchial metastases due to prostate cancer, however, are exceedingly rare. The occurrence and diagnosis of most endobronchial metastases usually follows the identification of the primary malignancy, with an average lead time of around 65 months as reported by Kiryu et al. to a median latency period of 136 months as reported by Marchioni et al. [3,4]. Simultaneous identification (synchronous) of the primary site and the endobronchial metastases is even more unusual with only 6% cases reported by Marchioni in his case series of 174 cases [4]. None of these synchronous cases, however, had prostate cancer although overall 5% of cases had endobronchial metastases with a prostate primary. In literature, only 3 synchronous cases of EM and prostate cancer have been reported in the last 15 years based on our PubMed search [Table 1]. We present a similar rare case with a synchronous reveal of endobronchial metastases secondary to prostatic adenocarcinoma.

**Table 1:** Case reports of synchronous EM & prostate cancer diagnosis.

Author	Year	Patient's age	Symptoms	Treatment	Extrapulmonary Metastases
Shen[5]	2008	72	None	Chemotherapy	Bones
Garai[6]	2010	84	Haemoptysis & Weight loss	Hormonal Therapy and Subcapsular Orchidectomy	Lung
Lee[7]	Not available	Not available	Not available	Chemotherapy	None
Our case	2019	61	Dyspnoea & Chest tightness	Hormonal Therapy, Chemotherapy (declined by patient)	Bones

## 2 Case

A 61 years old Egyptian gentleman presented with a 6 months history of progressive exertional dyspnoea associated with chest tightness to the Emergency Department. He had been treated 2 months earlier for a Non-ST elevation myocardial infarction, when he presented with similar symptoms and had a troponin rise from 28 to 81 and a computerised tomographic coronary angiogram showed moderate to severe right coronary artery and mild left anterior descending artery disease. Other symptoms on this occasion included a productive cough and a significant unintentional weight loss over the past 6 months. He did not have any other previous medical problems, and he did not have any occupational exposures of note. His height was 165cm with a weight of 74 kg. He was afebrile on admission with a heart rate of 99 beats per minute, blood pressure of 126/77 mmHg and oxygen saturation of 93% on air with a respiratory rate of 23. He had bilateral temporal muscle wasting but no clubbing, palpable lymphadenopathy or peripheral oedema. Chest auscultation revealed bilateral coarse crackles up to mid‐zones. His blood work showed a white blood cell count of 4.0 × 10^3^/μl, haemoglobin 11.3 gm/dl, MCV 80fl, alkaline phosphatase of 306 U/L with normal calcium, renal and liver function tests. A chest X-ray revealed airspace opacities bilaterally in the mid and lower zones and diffuse sclerotic changes in the visualised bones. Sputum was negative for acid-fast bacilli and general microbiology on smear and cultures.

**Fig. 1:**
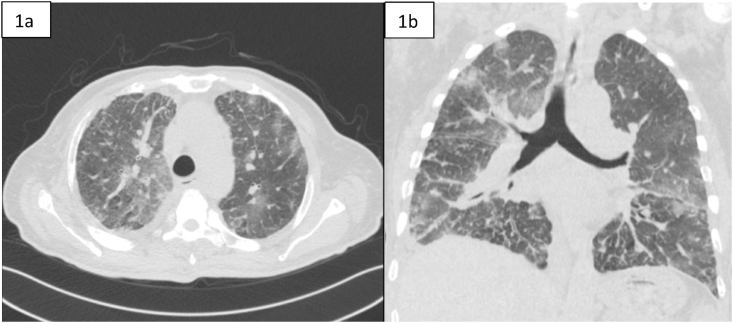
a & 1b: CT chest showing bilateral diffuse reticulonodular ground glass changes but 1a no obvious EM 1b.

Computed tomography (CT) of the chest showed diffuse reticulonodular and ground-glass opacities involving both lung fields associated with interlobular septal thickening but no apparent EM [Fig. 1a,b]. The lung changes suggested a chronic pneumonitis type picture, nevertheless, the possibility of metastatic nodules/disease could not be excluded. Diagnostic bronchoscopy showed multiple tiny endotracheal and bronchial nodules in the anterior wall of the distal trachea and major bronchi [Fig. 2a, Fig. 2ba and b]. Histology of the endobronchial nodules on biopsies showed bronchial mucosa heavily infiltrated by a tumour characterized by cords, vague nests and vague acini consisting of small-sized tumour cells, some having hyperchromatic nuclei with prominent nucleoli [Fig. 3a, Fig. 3ba].

**Fig. 2a:**
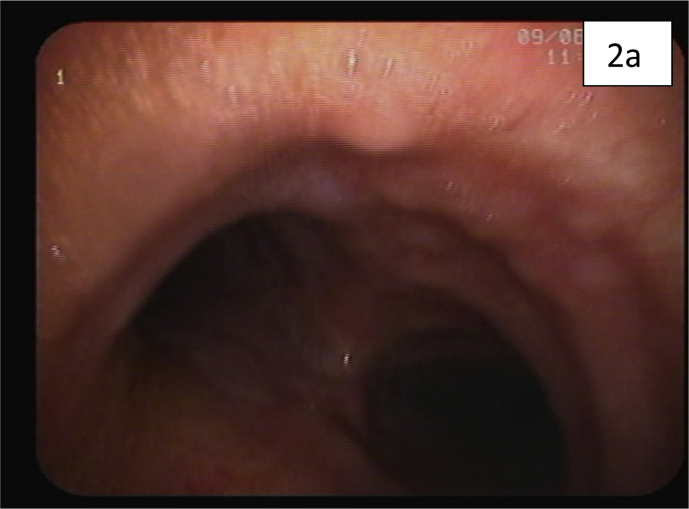
Endotracheal metastatic nodules anteriorly just above the carina.

**Fig. 2b:**
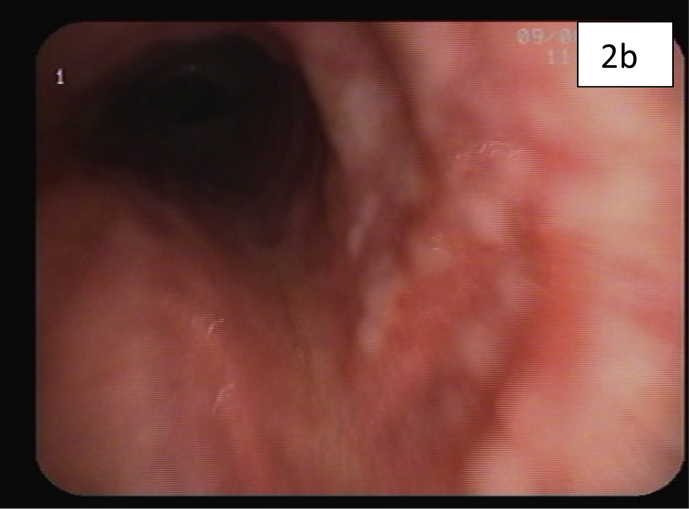
Left main bronchus showing endobronchial metastatic nodules 2a 2bA.

**Fig. 3a:**
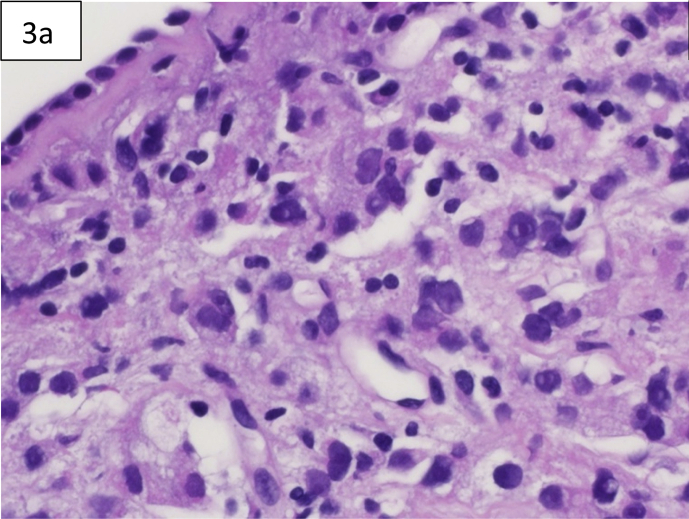
H&E: Showing tumour cells underneath the atrophic bronchial mucosa(x600).

**Fig. 3b:**
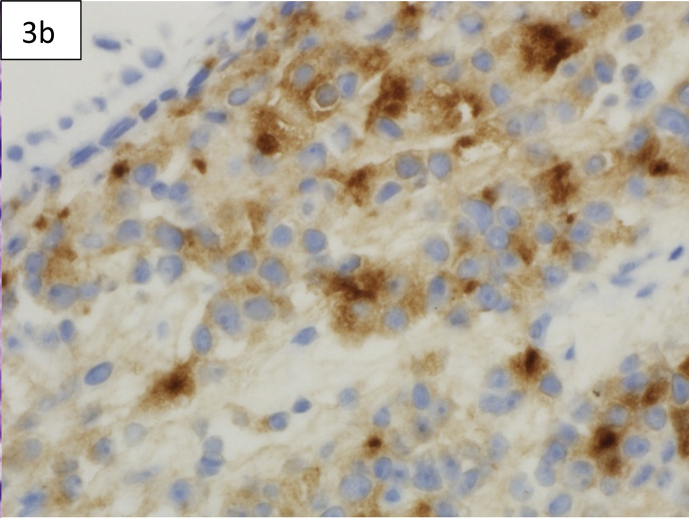
Immunohistochemistry for PSA positive in tumour cells and negative in bronchial cells(x600) 3b 3a.

Immunohistochemistry was strongly positive for CK AE1/AE3 and PSA confirming metastatic prostatic adenocarcinoma [Fig. 3b]. CT of abdomen and pelvis revealed a diffusely heterogeneous and enlarged prostate with postero-lateral bladder wall thickening and prominence of lymph nodes within the abdomen and pelvis. Coupled with this were extensive sclerotic bony lesions. His total prostate-specific antigen (PSA) was more than 5000 ng/ml. A bone scan showed multiple deposits involving the ribs, vertebrae, sternum, clavicles, humeri, femora, skull and the bony pelvis. Subsequently, he was started on hormonal treatment by the Uro-oncology team with his last PSA dropping to 62 ng/ml. He was offered chemotherapy as well with Docetaxel which he declined.

## 3 Discussion

Most endobronchial lesions are due to primary lung cancers. Lungs are, however, one of the commonest sites of metastases from solid extrapulmonary tumours, but these metastases presenting as endobronchial growths is very unusual. Frequency estimates for EM are from relatively old, largely autopsy based case series. King and Castleman were the first to demonstrate, the frequency of endobronchial involvement in 18% of their 109 patient autopsy series [8]. Braman and Whitecomb, however, reported a 2% involvement of bronchi in their autopsy series of 244 [9]. There is a wide variance in frequency from 2 to 50% across different studies based on the definition used for endobronchial involvement [4]. The epidemiology of EM is therefore still very unclear. Although the growing use of CT scans and bronchoscopies is likely to be useful in clarifying the epidemiological mist surrounding EM in the future. In his 2014 study in this context, Marchioni et al. documented endobronchial metastases in 4% of all bronchoscopic procedures performed for suspected malignancies [4].

In his study of 204 patients, Sorensen et al. noted that the majority (148/204) had extrapulmonary metastases at other sites at the time of diagnosis of their EM [10]. We also found bone metastases simultaneously in our case. Clearly, the diagnosis is easier to pin down in such cases, but it is more difficult when EM are the only sign of either an initial presentation or recurrence of an extrapulmonary malignancy especially given long latency periods. Given the huge variance in frequency of EM, it is also possible that there are some missed clinical diagnosis and a possible underreporting in the literature of such cases.

The most common symptoms and radiological findings (Table 2) due to EM are similar to those of primary bronchogenic carcinomas and hence unless the primary malignancy is apparent on cross-sectional imaging, a diagnosis is difficult without a bronchoscopy and histopathological and immunohistochemical analysis of biopsies.

**Table 2:** Most Common symptoms & radiological features of EM from the two largest case series.

SYMPTOMS	Sorenson et al.[10]	Marchioni et al.[4]
Cough	48%	22%
Dyspnoea	37%	17%
Haemoptysis	37%	12%
Chest pain	7%	Not available
Asymptomatic	20%	24%
RADIOLOGICAL FEATURES		
Normal	4%	Not available
Pleural effusion	8%	23%
Visible tumour	26%	Not available
Multiple Nodules	17%	53%
Atelectasis	58%	23%
Mediastinal adenopathy	4%	47%

Kiryu et al. suggested an EM development model consisting of four EM types. Type 1 due to direct bronchial metastases, type 2 due to bronchial invasion of a parenchymal lesion, type 3 due to bronchial invasion of mediastinal or hilar nodal metastases, and type 4 due to the extension of the peripheral lesion along the proximal bronchus. He discusses median survival times based on these developmental types as well, but the difference was statistically important only between type 3 (2 months) and type 4 (18 months). Survival, in general, is poor as the diagnosis of EM normally represents more advanced disease but depends on the biological behaviour of the tumour in question as well. In his study Heitmiller et al. reported a mean survival of 12.5 months, Ettensohn et al. of 21 months and Baumgatrner et al. of 32 months only [[11], [12], [13]].

The suggested treatment of EM secondary to prostate cancer is hormone therapy, however surgical resection of the metastasis and palliative treatment with brachytherapy is also used [[14], [15], [16], [17], [18]]. The treatment is also dependent on the biological status of the disease, as androgen refractory disease does not respond to standard hormonal treatment and requires chemotherapy. Thus, treatment options have to be personalised based on these factors, performance status and the patient's wishes.

## 4 Conclusion

Endobronchial metastases secondary to prostate cancer are a rare occurrence. Synchronous diagnosis of the primary malignancy and EM is even rarer. Bearing in mind the long latency period, patients with seemingly non-specific respiratory symptoms of cough, dyspnoea and chest pain and or unexplained weight loss, but with a confirmed current or previous diagnosis of an extrapulmonary malignancy associated with a higher risk of endobronchial metastases should be considered for a bronchoscopy. Bronchoscopy in conjunction with immunohistochemical analysis is the key to clinching the diagnosis in such cases.

## Declaration of competing interest

The authors declare that they have no known competing financial interests or personal relationships that could have appeared to influence the work reported in this paper.
